# Book Reviews

**Published:** 1803-01-01

**Authors:** 


### CRITICAL ANALYSIS

OF THE

### RECENT PUBLICATIONS

ON THE

### DIFFERENT BRANCHES OF PHYSIC, SURGERY, AND MEDICAL PHILOSOPHY.

---

*The Principles of Surgery*; *in two volumes. Volume First*, *of the ordinary Duties of the Surgeon*, *&c. Volume Second*, *a System of Surgical Operations*, *&c. By* JOHN BELL, Surgeon. Edinburgh, 1801, pp. 674, 4to.

THOUGH only the first volume of this work has come to our hands, we shall no longer delay to direct the notice of our readers to a publication which offers such a singular variety of interesting matter, so much entertainment, so much excellent sense, and such ample food for criticism.

Our limits and plan, however, only allow of *analysis*; and amidst such a profusion of desultory matter, and (we may add) a *display* of professional learning, the task of analysis is in no small degree perplexing.

The object of the work before us is the excellent and laudable one of forming the accomplished Surgeon; of exhibiting to the student all the variety of knowledge requisite to render him a thorough master of every branch of his profession; equal to all emergencies; great in little things as well as in objects of magnitude; able to rely upon his own resources and ingenuity; and ready to act at all times in the exercise of a profession which demands, in a peculiar manner, the rare alliance of prompt decision with prudent caution.

The volume opens with a short preliminary Discourse on the education and duties of a Surgeon; and it would be injustice to our author not to add, that he has seized upon the precise points to which the attention of the young surgeon should be directed, and with excellent good sense he notices some of the prevailing errors in surgical education. One of the most important of these errors is an overweening love for the shewy and splendid branches of surgery: "Such," the author observes, "is the natural horror of blood, and the hesitations and difficulties of the surgeon himself, when any thing so daring as a dangerous operation is to be done, and such are the unceasing and anxious inquiries of friends, that operations, though the least part of our profession, strike a deeper interest into the public mind than the daily cures we perform. Operations usurp an importance in Surgical Education, which they should not naturally naturally have. Operations have come at last to represent the whole science; and a surgeon, far from being valued according to his sense, abilities, and general knowledge, is esteemed excellent only in proportion as he operates with skill."

Indolence is another great bane to professional excellence, which the author describes (we are afraid too justly) as a fault peculiarly applicable to modern education; to those who learn the most arduous of all sciences " by hearsay and report, by walking the wards of hospitals, lounging through the classes of a college, and by hearing and repeating the news of the passing day." The qualities of a surgeon required by Celsus, *manu strenua stabili, nec unquam intremiscente, animo intrepidus immisericors* (a hand, firm, steady, and untrembling; a mind, bold and merciless) are ill engrafted upon the mild humane feelings of the well educated gentleman; and it is not without reason that the author contrasts the often assumed character of boldness in operations, with the calm steadiness arising from accurate knowledge and practised skill.

In the second Discourse, the author enters upon his subject by a view of the doctrine of adhesion, particularly as it applies to the treatment of recent wounds. As it is his object to compare and connect the antient with the modern practice, he describes the method of treatment pursued by the older surgeons, and their dreadfully bad custom of plugging up every fresh wound with tents, and of taking a variety of other means to convert every recent wound into a suppurating sore. The illustrious Taliacotius appears to have the merit of introducing some of the first improvements in this important branch of surgery; but through the extravagance of some of his followers, and the irresistible operation of the wit and humour of his satirists, his name has become irrevocably connected with ideas of ridicule.

The practice of procuring adhesion or union by the first intention, our author very justly observes, " has done more for Surgery in a few years than any other general observation, not excepting even the greatest of all discoveries, the circulation of the blood." Though this valuable practice has made its way by imperceptible gradations, and through the efforts of a number of eminent men, it is indebted more particularly, as the author remarks, to Mr. Hunter and the London school.

The chapter on sutures is clear, precise, and more entirely practical than often occurs. " Surgeons," he observes, " have practised all imaginable methods of uniting wounds, and very proud have they been of their sutures, and still prouder of the names they have given them." The rules which are laid down for the reunion of deep muscular wounds, and the earnestness with which the importance of procuring speedy adhesion is inculcated, highly merit the attention of the student.

The third Discourse treats of the nature of ill-conditioned and complicated wounds, of ulcers, dressings, bandages, and the daily duties of an hospital surgeon. The reader must be aware what an important, important, extensive branch of surgery is included in this chapter, and how large a portion it includes of the daily duties of the busy, harassing, and actively useful life of the camp or navy surgeon. We have no hesitation in recommending the whole of this chapter to our readers, as replete with admirable sense and sound doctrine; and, without involving the subject in tedious and unmeaning classification, the author, in following the full and descriptive mode of instruction which particularly suits the class-room, has thrown together a series of observations which every student will read to advantage.

A highly proper and we fear very generally necessary precaution is given concerning the subject of poultices. As this application usually falls to the province of the nurse rather than the practitioner, it is much to be feared that it is too often resorted to as an excuse of indolence or incapacity. "I must truly confess," says our author, "that while we are improved in all the great and difficult parts of surgery, we are gone backwards in all the nice and delicate attentions which are so necessary in the cure of wounds. Long ago, a surgeon could never do too much in the way of probing and searching wounds, tenting them to the quick, injecting them with balsams, and torturing the limbs with injudicious bandages; but now, a surgeon thinks he has done enough in clapping a plaster over a sore with the palm of his hand, or with clean hands he feels the pulse for form's sake, and orders the limb, without regard to its condition, to be laid in a mash of poultice." Instead of laying a relaxed and extensively suppurating limb in a mash of poultice, where the patient for half an hour's warmth and comfort suffers permanent cold, filth, and moisture, how much better and more consonant to the soundest practice is the following method, which we cannot forbear giving in the author's words. "We remove the poultice, and lay a piece of dry lint on the wound; we use a stimulant fomentation made of a strong decoction of camomile flowers, with sal ammoniac and spirits, to cleanse and excite the skin; we dry the limb thoroughly, wrap it up in warm flannel, and remove the foul and moist clothes; we dress the wound the next day with slips of plaster, that the dressings may come easily off, and with soft and fine lint fill up the basis of the sore, laying a flat sponge above the lint to absorb the matter of the wound. Let the lips of the wound be supported with soft pledgets of lint, and the whole limb be rolled carefully from the toes upwards, &c."

Activity and vigour are the characteristics of the practice which our author lays down, such as a frequent use of the lancet in giving a speedy outlet to all abscesses within the muscular or deep-seated parts, a constant recourse to all the auxiliaries of pressure, stimulants, and astringents, and all the mechanical means that the contrivance of the surgeon can suggest.

Under the treatment of complicated wounds, the author considers the subjects of ulcers, hospital gangrene, and gun-shot wounds; and he cites the history of several valuable cases of our own brave, thoughtless, and intemperate seamen, wounded in various actions of of the late war, who have afforded striking examples of every degree of these dreadful calamities. These are subjects which peculiarly interest the hospital or military surgeon, but an acquaintance with them is highly requisite to all practitioners.

In the next Discourse, the author notices the important practical topics of bandages, and gives an elegant selection (illustrated with plates, as in all the other parts of the work) by the judicious use of which every part of the body may undergo the benefits of this application. It is sufficient to observe, that this selection is judicious; and the importance of bandages, in a practical view, draws from the author another censure of the carelessness to every-day objects, and the neglect of little attentions which so often characterises modern surgical education.

In the fifth Discourse, the author gives an historical view of the various means employed from the earliest periods of surgery to modern times, of preventing the most alarming of all accidents, *haemorrhagy.* He observes, with great force, that "it is the dashing of the blood from the great arteries, and the fainting of the patient, that hurries our most important operations, and makes all the difference between operating on the living body, and dissecting the dead. It is this which unsteadies, at times, the hands of the boldest surgeon, and makes his heart at the first alarm sink within him. No surgeon or spectator can keep the natural colour of his cheek when a patient is expiring by loss of blood; and the actual death of a patient must leave a lasting melancholy on the surgeon's mind."

The History of Science hardly affords any more satisfactory example of gradual and high improvement than in the treatment of hæmorrhagy. From the dreadfully absurd and superstitious incantations, and monstrous rites, of the antients; from the cruel (though often powerful) use of the actual cautery, and the boiling oils and turpentines; from the supremely ludicrous follies of the sympathetic powders and washes, introduced by Kenelm Digby, and other alchemical quacks of the same stamp; how great is the progress of improvement to the needle and ligature of Paré; to the judicious use of mechanical styptics and continued pressure! We shall not attempt to analyse rigorously the contents of this historical view of the subject, though curious and entertaining, but shall only touch upon a few of the author's observations on the modes of suppressing hæmorrhagy actually in use. The illustrious Petit was the last advocate of eminence for the practice of compression, used in its most extensive application; and having employed much perverted ingenuity in explaining the circumstances of suppression of hæmorrhagy, from a wounded artery, by the supposed shape of the clot or coagulum formed above the separation of the vessel, he applied his theory to practice, and introduced the dangerous method of attempting to stop hæmorrhages of large arteries by compression alone. This fallacious theory of the shape of the coagulum has long been exploded. The contraction of the artery through its whole extent, extent, from the divided extremity up to its nearest inosculating branch, is another opinion supported by White, Kirkland, and Aitken. The explanation of the natural causes by which hæmorrhagy is stopped, is however explained by our author, on the principle of the surrounding cellular substance being injected with blood flowing from the divided artery, which extravasation is, in many cases, a sufficient barrier to restrain the bleeding till the parts inflame, and the artery is entirely stopped. Pressure, therefore, according to our author, only restrains the open flow of blood, but does not prevent it from insinuating itself into the adjacent cellular substances; and hence, he argues, when any sudden displacing of parts, any exercise or motion is made in the newly-closed wound, the cellular compress is removed, and the artery bleeds afresh. The whole of the author's explanation is illustrated by the not unfrequent, and often very perplexing accident of a wound in the radial artery; and in whatever manner the explanation of facts may strike the reader, the description of this kind of accident, with the various modes adopted in order to stop hæmorrhage, will be perused with great satisfaction and advantage.

An inquiry of high importance, arising immediately from the preceding observations, is next considered. It is, *the condition of the artery in Aneurism, and the effects of ligatures upon the artery.*

After relating the histories of several cases from various surgical writers of aneurism of the brachial artery arising from accidents in venæsection, he considers the question, whether the compression usually employed in these instances effects a cure by uniting the sides of the wound in the artery leaving it pervious, or by obliterating the vessel altogether, and leaving the circulation to be continued through the inosculating branches. Most Anatomists will, (we believe) agree with the Author in adopting the latter opinion. The effect of two ligatures passed round an artery, so as to intercept a portion of the vessel between them, is another point of importance: they operate "by making the several points of the arterial canal pass through the several stages of inflammation, from adhesion in one point to gangrene in another." The space included between the ligatures falls into gangrene; the space immediately under the stricture of each ligature adheres; this adhesion prevents the gangrene or inflammation from passing along the higher parts of the arterial canal; but the inflammation affects the arterial tube a little way upwards and downwards, so as to thicken its walls and contract its cavity, whence the canal of the artery is obliterated a little way beyond the exact place where it is tied." This explanation is illustrated by a very perspicuous drawing.

The cause of the secondary hæmorrhagy, which has so frequently defeated the object of the modern operation for aneurism, being, as our Author observes, in almost every case, *ulceration of the artery*, a number of practical observations are deduced, relating to the method of applying the ligature, the time of withdrawing it, and the degree to which the artery is to be exposed during the operation.

[ *To be continued.* ]

*Essays on the Diseases of Children, with Cases and Dissections; by* JOHN CHEYNE, M. D.—*Essay* 1. *On Cynanche Trachealis, or Croup.* Edinburgh.

THE diseases of children form a very interesting, and in some instances almost a peculiar branch of medical practice. The character of the infantile constitution, which operates more or less whatever other habit of body may be present, and gives a tendency to peculiar diseases; the age of the subject, which prevents the practitioner from receiving much advantage from his patient's own description of symptoms; and the domestic economy of the nursery, which most powerfully promotes, in some instances, and counteracts in others, the plan of the Physician;—all these circumstances contribute to give a peculiar character to the investigation of the diseases of children. The Author expresses it to be his design "to discuss, in separate Essays, the most important of the diseases of children, beginning with those, as less intricate, to which children, after being weaned, are exposed, and proceeding afterwards to those which attack infants at the breast."

This order is the contrary of that which we should naturally expect; but in detached Essays, each unconnected (if we may judge by the specimen before us) with the succeeding, the Author may be allowed to consult his own convenience.

He could not have selected a more interesting subject than the present; for the Cynanche Trachealis, or Croup, is a disorder of the highest importance to the practitioner to be so familiar with as to be able to detect very early in its progress, on account of its extremely dangerous nature, and the necessity of immediately having recourse to the most vigorous measures for its removal.

After a very concise history of the disease, the author gives an accurate description of the symptoms and the pathology. No new conjectures are added concerning several of the difficult points in the pathology of Croup; one circumstance, however, is explained in the following manner: "There is a circumstance mentioned in the history of the disease, which I have not seen satisfactorily resolved: I allude to the sudden extinction of our hopes when they are at the highest, consisting, first, in a wonderful remission of the disorder, and soon after in a fatal exacerbation. Perhaps this ought to be attributed rather to a mechanical than to a spasmodic affection of the parts. It sometimes takes place after the expectoration of part of the membrane; and I suppose that the connection of the remainder with the trachea may be loosened; so that, in taking a full inspiration, this detached portion acts as a valve, compleatly shutting up the tube, and thus suddenly suffocating the child."

Nothing peculiar occurs in the mode of treatment. The author with justice places his chief confidence in blood-letting, either from the arm, or in preference, from the jugular veins, or else by leeches.

The following facts concerning the causes of the disease merit attention: "I have observed that some families are much more liable to this complaint than others; very often, when one child in a family takes the disorder, the other children are sooner or later affected in a similar way. In the second place I have observed, that in Leith the danger is greater or less in exact proportion to the nearness or distance from the sea-shore; and I conclude, that the observation would hold good elsewhere. Of all the instances I have seen of the disease this year, amounting to ten or eleven, not one of the children lived a stone's throw from the sea-shore or harbour. In Edinburgh, which is only a mile and a half distant from the sea; nay, in the skirts of Leith, the farthest from the beach, although not a quarter of a mile removed, the disease is rare."

The greater part of the volume is occupied with the history of several very illustrative cases, to which are subjoined five beautiful and accurate coloured plates, that show, in a very perspicuous manner, the nature and appearance of the parts concerned in, and changed by, this dreadful disease, a reference to which cannot fail of immediately making the reader familiar with this important example of Morbid Anatomy.

The Author endeavours to distinguish Croup from the acute Asthma, described by Miller, by the following diagnostics: "In Croup the cough is constantly ringing in our ears; in the acute Asthma there is no cough. In Croup there is very seldom any remission; the remission in acute Asthma is one of the most striking phenomena of the disease, and it is attended with some evacuation, as belching, vomiting, or purging. In Croup, the pulse is strong, the urine high coloured, the fever is much greater, the voice sharp and small; in acute Asthma, the pulse, though perhaps equally quick, is less full, the urine is limpid, and the voice is croaking and deep."

The distinction between these two diseases is the more important as the practice to be pursued is different, at least so far that the antiphlogsitic method, and bleeding in particular, is indispensable in Croup, but of very doubtful efficacy in the Asthma.

---

*An Apology for differing in Opinion from the Authors of the Monthly and Critical Reviews; on the Subject of Variolous and Vaccine Inoculation; on Dr. Jenner's Discovery; on the Means of preventing Febrile Contagion; and on the Establishment of Charitable Institutions; by* J. C. Lettsom, M. D. & LL. D. octavo, pp. 60. London, 1803.

The practical opinions and facts contained in this pamphlet are so consonant to those we have always inculcated, that we have no hesitation in expressing our approbation of it.

